# Normative values of muscle strength across ages in a ‘real world’ population: results from the longevity check‐up 7+ project

**DOI:** 10.1002/jcsm.12610

**Published:** 2020-11-04

**Authors:** Francesco Landi, Riccardo Calvani, Anna Maria Martone, Sara Salini, Maria Beatrice Zazzara, Matteo Candeloro, Hélio José Coelho‐Junior, Matteo Tosato, Anna Picca, Emanuele Marzetti

**Affiliations:** ^1^ Center for Geriatric Medicine (CEMI) Fondazione Policlinico Universitario ‘Agostino Gemelli’ IRCCS Rome Italy; ^2^ Università Cattolica del Sacro Cuore Institute of Internal Medicine and Geriatrics Rome Italy; ^3^ Dipartimento di Medicina e Scienze dell'Invecchiamento Università G. D'Annunzio Chieti Italy

**Keywords:** Handgrip strength, Chair‐stand test, Sarcopenia, Screening, EWGSOP2, Early diagnosis

## Abstract

**Background:**

Low muscle strength is a powerful predictor of negative health‐related events and a key component of sarcopenia. The lack of normative values for muscle strength across ages hampers the practical appraisal of this parameter. The aim of the present study was to produce normative values for upper and lower extremity muscle strength across a wide spectrum of ages, in a large sample of community‐dwellers recruited in the Longevity check‐up (Lookup) 7+ project.

**Methods:**

Lookup 7+ is an ongoing project that started in June 2015 and conducted in unconventional settings (i.e. exhibitions, malls, and health promotion campaigns) across Italy with the aim of fostering the adoption of healthy lifestyles in the general population. Candidate participants are eligible for enrolment if they are 18+ years and provide written informed consent. Upper and lower extremity muscle strength is assessed by handgrip strength and five‐repetition chair‐stand [5 × sit‐to‐stand (STS)] tests, respectively. Cross‐sectional centile and normative values for handgrip strength and 5 × STS tests from age 18 to 80+ years were generated for the two genders. Smoothed normative curves for the two tests were constructed for men and women using the lambda‐mu‐sigma method.

**Results:**

From 1 June 2015 to 30 May 2019, 11 448 participants were enrolled. The mean age of participants was 55.6 years (standard deviation: 11.5 years; range: 18–98 years), and 6382 (56%) were women. Normative values for handgrip strength and the 5 × STS test, both absolute and normalized by body mass index, were obtained for men and women, stratified by age groups. Values of upper and lower extremity muscle strength across ages identified three periods in life: an increase to peak in young age and early adulthood (18–24 years), preservation through midlife (25–44 years), and a decline from midlife onwards (45+ years).

**Conclusions:**

Our study established age‐specific and gender‐specific percentile reference values for handgrip strength and the 5 × STS test. The normative curves generated can be used to interpret the assessment of muscle strength in everyday practice for the early detection of individuals with or at risk of sarcopenia.

## Introduction

During the last two decades, sarcopenia has received increasing attention by researchers and clinicians, such that it is now recognized as a ‘true’ disease with its specific International Classification of Diseases (ICD)‐10 code.^1^ In 2019, the European Working Group on Sarcopenia in Older People 2 (EWGSOP2) released an updated consensus on the definition and diagnosis of sarcopenia, identifying muscle strength as the main domain for its evaluation and assessment.^2^ As such, dynapenia, as determined by handgrip strength and/or the five‐repetition chair‐stand [5 × sit‐to‐stand (STS)] test, is now recognized as an indicator of probable sarcopenia.

Muscle strength remains substantially constant during the first decades of adulthood, starts declining at middle age (45–50 years), and drops remarkably in late life.^3^, ^4^ As a result, people aged 70 years experience a 30% loss of muscle strength relative to their peak values.^3^, ^4^ Cut‐offs for handgrip strength and 5 × STS tests indicated by the EWGSOP2 identify the likelihood of being sarcopenic in older persons only and cannot be applied to the adult population or younger adults. Muscle mass and strength in advanced age are largely influenced by peak values attained during adulthood.^5^ Indeed, while sarcopenia is in essence a condition of old age, its development is often the consequence of constitutive factors as well as long‐standing lifestyle habits and diseases.^2^ This implies that people at risk of becoming sarcopenic might be characterized by lower‐than‐normal values of muscle mass and strength during adulthood and midlife. However, no conclusive normative data for muscle strength across ages are presently available, which hinders the possible identification of at‐risk individuals before declines in muscle strength have reached a critical threshold. Dodds and colleagues^6^ published normative values of muscle strength by assembling data from 12 different studies in Great Britain for which information on handgrip strength was available. Yet, included studies were heterogeneous in terms of sample size, age, gender distribution, and protocols for assessing handgrip strength. Furthermore, the investigation focused on handgrip strength only, and no indications on lower extremity muscle strength were provided. Handgrip strength is generally considered to be a surrogate for overall muscle strength^2^, ^7^; however, its agreement with lower extremity muscle strength has recently been questioned.^8^ Moreover, lower limb performance was found to be related to incident disability more closely than handgrip strength in a large sample of older women.^9^ Upper and lower extremity performance tests, therefore, provide complementary information on functional status.^10^ Indeed, EWGSOP2 recommends either the handgrip strength or the 5 × STS test for the initial assessment of sarcopenia.^2^ The choice of a specific test should be based on the availability of equipment and trained assessors and the characteristics of the test person.^7^

The aim of the present study was to produce cross‐sectional centile values for handgrip strength and 5 × STS tests across ages, using a large sample of community‐dwellers enrolled across Italy in the Longevity Check‐up 7+ (Lookup 7+) project.^11^ Although the study was designed before EWGSOP2 release, the protocols followed for both handgrip strength and 5 × STS tests were compliant with the EWGSOP2 recommendations.^2^

## Materials and methods

Data used in the present study are from the Lookup 7+ project, an initiative promoted by the Department of Geriatrics of the Fondazione Policlinico ‘Agostino Gemelli’ IRCCS at the Università Cattolica del Sacro Cuore (Rome, Italy).^12^ The Lookup 7+ project was designed with the aim of promoting the adoption of healthy lifestyles in the general population. People visiting public spaces (i.e. exhibitions and shopping centres) or those adhering to prevention campaigns launched by our institution were recruited. Small (<100 000 inhabitants), medium (100 000–250 000 inhabitants), and large cities (>250 000 inhabitants) were chosen to have a comprehensive geographic coverage of mainland Italy and major islands. Places were selected based on the number of candidate participants who could be approached. In major cities (i.e. Rome, Milan, Naples, Genoa, Bologna, and Catania), activities were conducted in different locations to allow sufficient representation of the sociodemographic characteristics of inhabitants. The Lookup 7+ protocol was approved by the Ethics Committee of the Università Cattolica del Sacro Cuore (protocol #: A.1220/CE/2011) and is described in detail elsewhere.^11^, ^12^ The manuscript was prepared in compliance with the STrengthening the Reporting of OBservational studies in Epidemiology guidelines for observational studies.^13^

### Study sample

Between 1 June 2015 and 30 May 2019, 11 448 persons were enrolled in different Italian cities adhering to the national Lookup 7+ project. Candidate participants were considered to be eligible for enrolment if they were at least 18 years old and provided written informed consent. Exclusion criteria were self‐reported pregnancy, inability to perform the physical performance tests (e.g. wheel‐chaired), or inability to give written informed consent. Analyses of the present study were conducted in 11 331 persons for handgrip strength analysis (after excluding 117 participants with missing handgrip strength measurement data) and 11 164 participants for 5 × STS analysis (after excluding 284 people with missing 5 × STS test measurement data). The general characteristics of the excluded participants did not differ from those with complete data for the variables of interest.

### Data collection

All persons who accepted to participate in the study were offered individual assessment consisting of a brief questionnaire and the objective measurement of several parameters. In particular, the Lookup 7+ visit was structured to collect the following information and data: informed consent, lifestyle habits (smoking and dietary habits and physical activity), and measurement of body mass, height, blood pressure, blood glucose, total cholesterol levels, and muscle strength.^11^, ^12^ Body mass index (BMI) was calculated as the ratio between body mass (kg) and squared height (m^2^).

### Muscle strength testing

Handgrip strength and 5 × STS tests were conducted according to the EWGSOP2 recommendations.^2^ For both tests, inter‐rater and test–retest reliability was assessed during the investigator training phase and was found to be consistent with values reported in literature.^7^ Trained investigators certified by the standardization team appointed for the ‘Sarcopenia and Physical fRailty IN older people: multi‐componenT Treatment strategies’ (SPRINTT) randomized clinical trial^14^ performed all evaluations. Upper extremity muscle strength was assessed by handgrip strength using the same model of dynamometer throughout the study (North Coast Hydraulic Hand Dynamometer; North Coast Medical, Inc., Morgan Hill, CA, USA). The measure was obtained while the participant was seated on a chair with the shoulder in a neutral position, the elbow near the trunk and flexed at 90°, and the wrist in a neutral position (thumbs up). The contralateral arm remained relaxed under the thigh. Participants performed one familiarization trial before testing. Handgrip strength was measured in both hands and the highest value (kg) was used for the analysis.^4^, ^15^

Lower extremity muscle strength was assessed via the 5 × STS test. Participants were asked to stand up from a chair with their arms folded across the chest five consecutive times as quickly as possible. A standard armless chair, 43–47 cm in height, was used. The chair's height was chosen to allow participants placing their feet comfortably on the floor and to minimize the influence of chair's seat height on the test results.^16^ The back of the chair was secured against a wall to ensure safety and stability. The time taken to complete the task(s) was measured using a handheld stopwatch and was used for the analyses.^17^, ^18^

### Statistical analysis

All analyses were performed using the SPSS software version 11.0 (SPSS Inc., Chicago, IL). Continuous variables are shown as mean ± standard deviation (SD), categorical variables as frequencies by absolute value and percentage of the total. Descriptive statistics were run to describe demographic and main clinical characteristics of the study population according to age groups and gender.

Smoothed percentile curves for handgrip strength and 5 × STS test values, both absolute and normalized by BMI, were constructed for men and women separately using the lambda‐mu‐sigma (LMS) method (LMS Chart Maker Pro Version 2.54, Medical Research Council, London, UK).^19^, ^20^ LMS allows skewed distributions to be transformed into normal distributions using the maximum likelihood method, adjusting the curves of median (M), the coefficient of variation (S), and the Box–Cox power (L), and smoothing percentile curves of handgrip strength and 5 × STS tests values by cubic natural smoothing spline functions.^20^, ^21^

## Results

The mean age of participants included in the analyses was 55.6 years (SD: 11.5 years; range: 18–98 years), and 6382 (56%) were women. The general characteristics of the study sample according to age and gender are summarized in Table 1. The proportion of smokers, including active and former smokers, increased progressively with age in both genders, with a greater prevalence in men in all age groups. BMI increased with age up to 65 years and declined slightly thereafter. In all age groups, BMI was higher in men than in women. Healthy diet—defined as the consumption of at least three portions (~400 g) of fruit and/or vegetables per day^22^—was more frequent in older age groups, with no significant differences between genders. Finally, the rate of participation in any kind of physical activity for at least two times per week was higher in younger age groups than among older persons. For all age groups, the engagement in physical activity was more frequent in men than in women.

**TABLE 1:** General characteristics of the study sample according to age groups and gender

	Age groups (years)													
Characteristics	18–24	25–29	30–34	35–39	40–44	45–49	50–54	55–59	60–64	65–69	70–74	75–79	80+	All
**Men**														
Smoking (active/former)	47 (32)	64 (45)	87 (46)	144 (53)	146 (39)	203 (44)	277 (46)	315 (48)	305 (55)	337 (60)	312 (61)	194 (58)	129 (59)	2,560 (51)
BMI (kg/m^2^)	23.5 ± 3.4	23.8 ± 2.9	24.6 ± 3.5	25.7 ± 3.7	25.9 ± 3.6	26.4 ± 3.7	26.4 ± 3.8	26.5 ± 3.7	27.0 ± 3.9	27.3 ± 3.9	27.1 ± 3.6	26.5 ± 3.4	26.2 ± 3.3	26.3 ± 3.8
Healthy diet	81 (55)	81 (57)	101 (54)	140 (51)	205 (55)	268 (58)	345 (57)	389 (60)	343 (62)	417 (75)	404 (79)	271 (81)	174 (80)	3,219 (64)
Physically active	109 (74)	99 (79)	122 (65)	147 (54)	210 (56)	249 (54)	346 (57)	364 (56)	313 (57)	311 (56)	302 (59)	196 (59)	121 (56)	2,889 (58)
**Women**														
Smoking (active/former)	49 (26)	91 (36)	85 (36)	116 (41)	130 (37)	230 (36)	358 (38)	370 (43)	349 (44)	319 (42)	205 (36)	132 (33)	55 (28)	2,489 (38)
BMI (kg/m^2^)	21.6 ± 3.5	21.7 ± 3.5	22.6 ± 4.0	23.2 ± 4.5	23.5 ± 4.5	24.6 ± 4.6	24.7 ± 4.4	25.0 ± 4.5	25.3 ± 4.3	26.0 ± 4.2	26.5 ± 4.6	26.3 ± 4.7	26.3 ± 4.3	24.9 ± 4.6
Healthy diet	110 (60)	152 (60)	133 (56)	185 (65)	224 (64)	428 (67)	650 (70)	644 (76)	636 (80)	631 (83)	455 (80)	319 (79)	146 (74)	4,713 (73)
Physically active	119 (65)	149 (59)	138 (58)	147 (51)	177 (51)	309 (48)	484 (52)	433 (51)	424 (53)	411 (54)	303 (53)	194 (49)	91 (49)	3,379 (52)

Data are shown as absolute numbers (%), except for body mass index (BMI), which is reported as mean ± standard deviation

Normative values for handgrip strength (kg) in men and women, stratified by age groups, are shown in Tables 2 and 3, respectively. Mean values ± SD and the 5th, 25th, 50th, 75th, and 95th percentiles are reported. Handgrip strength values normalized by BMI in the two genders are listed in Tables S1 and S2. Muscle strength is similar in men and women during adolescence, after which men begin to gain strength more rapidly to a peak of 49 kg (±10 kg) around age 24, while in women, a peak of 29 kg (±5.6 kg) is reached at around age 29. Average handgrip values remain substantially stable up to age 40–44 and decline thereafter in both genders.

**TABLE 2:** Normative values for handgrip strength (kg) in men, stratified by age

Age groups (years)	Observations (*n*)	Centiles					Mean (standard deviation)
		5th	25th	50th	75th	95th	
18–24	146	30.0	38.0	44.0	50.0	61.0	44.5 (9.1)
25–29	139	30.0	40.0	44.0	50.0	59.0	44.8 (9.2)
30–34	186	30.0	40.0	46.0	51.0	61.3	45.6 (9.1)
35–39	270	32.0	40.0	46.0	52.0	62.0	46.3 (8.7)
40–44	369	31.0	42.0	47.1	52.0	60.5	46.7 (9.2)
45–49	455	31.0	40.0	46.0	52.0	60.0	45.6 (8.8)
50–54	598	30.3	39.6	44.6	50.0	59.3	44.4 (8.7)
55–59	639	30.0	38.0	42.0	48.0	56.0	42.7 (8.3)
60–64	547	26.8	35.0	40.0	46.0	62.0	40.1 (8.1)
65–69	552	27.0	34.0	38.0	42.0	51.0	38.3 (7.4)
70–74	503	22.4	31.2	35.1	40.0	48.0	35.6 (7.5)
75–79	329	20.5	29.0	32.7	38.0	44.5	32.9 (7.3)
80+	208	16.0	23.0	29.0	32.9	42.0	28.6 (7.9)
All	4,949	26.0	34.0	41.2	48.0	57.0	41.1 (9.6)

**TABLE 3:** Normative values for handgrip strength (kg) in women, stratified by age

Age groups (years)	Observations (*n*)	Centiles					Mean (standard deviation)
		5th	25th	50th	75th	95th	
18–24	181	16.2	22.0	27.0	31.8	37.9	27.5 (6.7)
25–29	248	19.0	24.0	28.0	31.0	38.0	27.7 (6.2)
30–34	234	18.0	23.3	27.0	30.1	40.0	27.6 (7.1)
35–39	280	20.0	24.0	28.0	32.0	38.0	28.0 (6.0)
40–44	345	20.0	24.0	28.0	31.0	38.0	28.1 (5.7)
45–49	633	18.0	23.0	26.0	30.0	38.0	27.0 (6.3)
50–54	919	17.0	22.0	25.1	29.0	35.0	25.6 (5.9)
55–59	839	16.0	20.0	24.0	28.0	34.0	24.4 (6.3)
60–64	786	14.0	20.0	22.0	26.0	32.0	23.0 (5.8)
65–69	758	14.0	18.0	22.0	24.0	30.0	21.8 (5.5)
70–74	563	10.0	16.0	20.0	23.0	29.0	19.8 (5.5)
75–79	395	10.0	14.0	18.0	22.0	27.0	18.1 (5.3)
80+	192	8.0	12.5	16.0	20.0	25.0	16.5 (5.8)
All	6,382	14.0	20.0	24.0	28.0	35.0	24.0 (6.7)

Normative values for the 5 × STS test(s) in men and women, stratified by age groups, are shown in Tables 4 and 5, respectively. Values of the 5 × STS test normalized by BMI in the two genders are listed in Tables S3 and S4. Mean values ± SD and the 5th, 25th, 50th, 75th, and 95th percentiles are reported. The best average performance on the 5 × STS test was observed at age 22 in women (6.0 ± 0.9 s) and at age 24 in men (6.0 ± 1.0 s). Similar to the handgrip strength test, the performance on the 5 × STS test remains substantially stable up to 40–44 years and declines thereafter in both women and men.

**TABLE 4:** Normative values for the 5‐repetition chair‐stand test (s) in men, stratified by age

Age groups (years)	Observations (*n*)	Centiles					Mean (standard deviation)
		5th	25th	50th	75th	95th	
18–24	146	4.7	5.3	6.0	7.0	8.6	6.3 (1.1)
25–29	136	4.7	5.6	6.3	7.6	9.1	6.6 (1.4)
30–34	186	4.7	5.5	6.6	7.5	9.0	6.7 (1.4)
35–39	268	4.8	5.9	6.7	7.8	9.0	6.8 (1.2)
40–44	367	4.4	5.7	6.8	7.8	9.6	6.8 (1.4)
45–49	625	4.9	6.0	7.0	8.1	10.0	7.2 (1.5)
50–54	910	5.1	6.3	7.3	8.5	10.6	7.5 (1.8)
55–59	830	5.1	6.5	7.7	9.0	10.9	7.8 (1.7)
60–64	772	5.8	7.0	8.0	9.1	12.0	8.3 (2.0)
65–69	747	6.0	7.2	8.2	9.9	12.7	8.7 (2.6)
70–74	547	5.8	7.7	8.9	10.4	13.7	9.2 (2.5)
75–79	377	6.6	8.1	9.6	11.1	15.9	10.1 (3.3)
80+	178	6.5	8.8	11.0	13.5	20.2	11.6 (4.1)
All	4,893	5.0	6.3	7.4	8.8	11.3	7.7 (2.1)

**TABLE 5:** Normative values for the five‐repetition chair‐stand test(s) in women, stratified by age

Age groups (years)	Observations (*n*)	Centiles					Mean (standard deviation)
		5th	25th	50th	75th	95th	
18–24	181	4.6	5.4	6.2	7.0	8.8	6.3 (1.3)
25–29	244	4.5	5.5	6.3	7.4	8.8	6.5 (1.3)
30–34	233	4.3	5.5	6.5	7.6	9.0	6.6 (1.4)
35–39	277	4.7	5.5	6.5	7.5	9.4	6.8 (1.5)
40–44	367	4.8	5.9	6.8	7.7	10.1	6.9 (1.5)
45–49	453	4.7	6.0	6.9	8.0	9.6	7.0 (1.4)
50–54	594	5.0	6.2	7.2	8.1	10.3	7.4 (1.9)
55–59	637	5.1	6.2	7.1	8.2	10.1	7.4 (1.6)
60–64	543	5.4	6.7	7.8	9.0	11.5	7.9 (2.0)
65–69	547	5.5	7.0	8.0	9.3	11.3	8.2 (1.9)
70–74	498	5.9	7.2	8.2	9.7	13.0	8.7 (2.3)
75–79	322	6.1	7.7	9.0	10.7	13.2	9.3 (2.3)
80+	189	5.9	8.2	9.6	11.2	14.9	10.0 (3.1)
All	6,271	5.0	6.4	7.7	9.0	12.0	8.0 (2.4)

**FIGURE 1:**
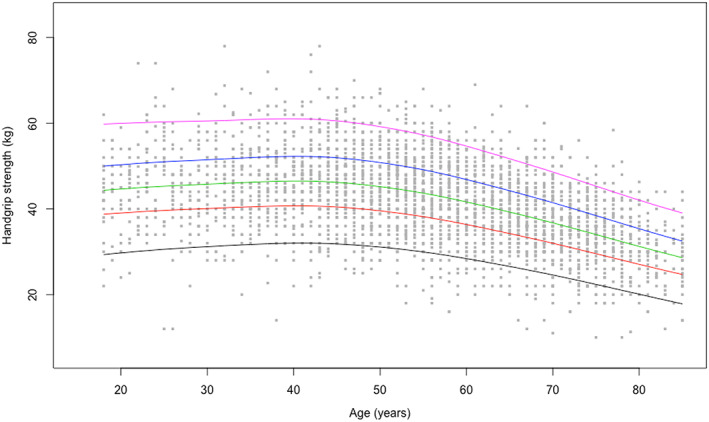
Handgrip strength reference percentiles for men aged 18 to 80+ years. The 5th, 25th, 50th, 75th, and 95th percentiles are shown in black, red, green, blue, and purple, respectively.

**FIGURE 2:**
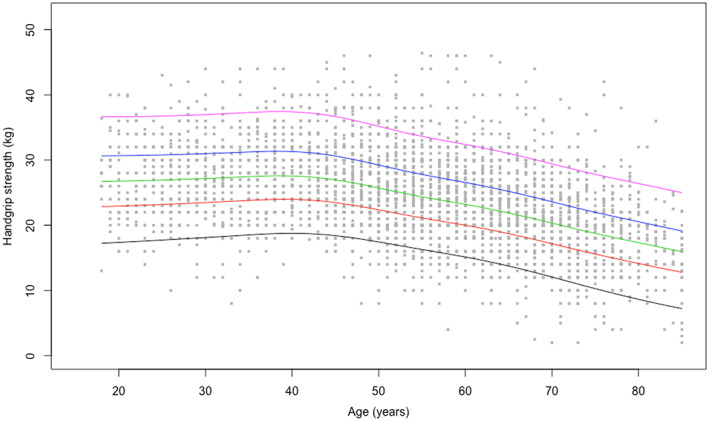
Handgrip strength reference percentiles for women aged 18 to 80+ years. The 5th, 25th, 50th, 75th, and 95th percentiles are shown in black, red, green, blue, and purple, respectively.

**FIGURE 3:**
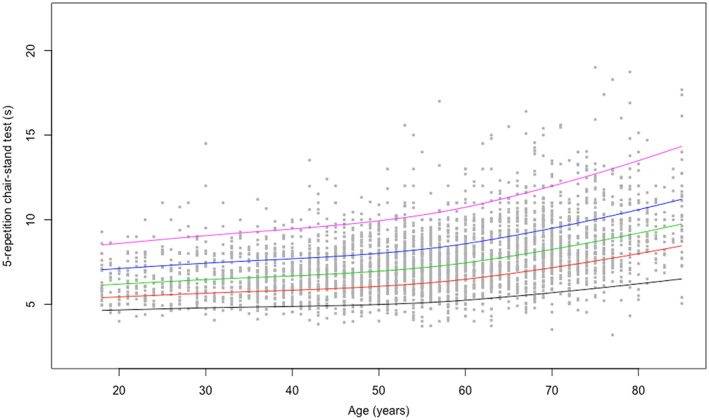
Five‐repetition chair‐stand reference percentiles for men aged 18 to 80+ years. The 5th, 25th, 50th, 75th, and 95th percentiles are shown in black, red, green, blue, and purple, respectively.

**FIGURE 4:**
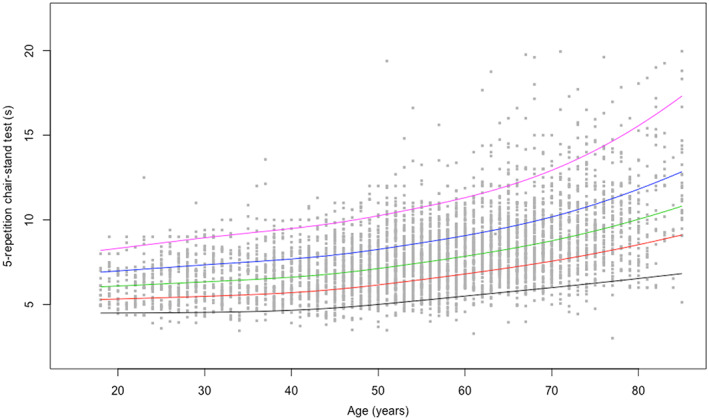
Five‐repetition chair‐stand reference percentiles for women aged 18 to 80+ years. The 5th, 25th, 50th, 75th, and 95th percentiles are shown in black, red, green, blue, and purple, respectively.

Centile values for handgrip strength and the 5 × STS test indicate three periods in life: an increase to peak in early adulthood (18–24 years), maintenance through midlife (25–44 years), and decline from midlife onwards (45+ years). To help translate these data in clinical practice, ad hoc charts (Figure 1 and 2 for handgrip strength; Figure 3 and 4 for the 5 × STS test) depicting the 5th, 25th, 50th, 75th, and 95th percentiles, from age 18 to age 80+, in men and women, were created. Charts with centile values for handgrip strength and 5 × STS tests normalized by BMI in the two genders are shown in Figures S1–S4.

## Discussion

Findings from the present study indicate that muscle strength, as assessed by handgrip strength and 5 × STS tests, increases to a peak in early adult life, followed by a period of maintenance throughout adulthood, prior to declining with advancing age. The normative values determined for handgrip strength and 5 × STS tests across age groups are instrumental for the practical interpretation of muscle strength measurements and the definition of thresholds for the identification of probable sarcopenia for use in clinical practice.^2^ Because sarcopenia has been proposed as the biological substratum of physical frailty and the pathway whereby the outcomes of physical frailty occur,^23^ our data may assist in the identification of people at risk of incurring negative health‐related events. This has considerable clinical and public health implications, because preventive interventions against sarcopenia and physical frailty may need to be implemented starting from middle age, when declines in muscle strength become clinically detectable. As previously reported, age‐specific and gender‐specific values falling below the 5th percentile for handgrip strength and above the 95th percentile for the 5 × STS test may be indicative of abnormally low muscle strength and should prompt additional investigation strength test.^24^ Alternative cut‐offs may be represented by the 25th percentile for handgrip strength and the 75th percentile for the 5 × STS.^25^

The present is the first large‐scale study to produce normative data for upper and lower extremity muscle strength across a wide age spectrum in an unselected study sample of Caucasian persons. According to the recently released EWGSOP2 sarcopenia assessment algorithm, the ‘entry point’ for the identification of people with probable sarcopenia involves the assessment of handgrip strength and/or the 5 × STS test.^2^ The pattern of changes of muscle strength across ages identified by our study is remarkably similar to the well‐established life course trajectory of bone mineral density.^26^ This supports the possibility of using muscle strength charts (Figures 1, 2, 3, 4) similar to what is routinely done with those describing children growth, BMI, and osteoporosis risk. Hence, charts produced by the present study may become a unique, simple and rapid tool for the assessment of all persons with or at risk of probable sarcopenia in any care setting.

Previous studies analysed trends of muscle strength across ages, with a special focus on childhood.^27^, ^28^, ^29^ To the best of our knowledge, only one study explored differences in muscle strength across a wide age range (4–90 years) in the general population.^6^ In this study, Dodds and colleagues^6^ combined 60 803 observations from 49 964 participants of 12 general population studies in Great Britain. Although offering interesting insights, findings were obtained through pooling data from heterogeneous cohorts in terms of sample size, age, gender distribution, and muscle strength assessment protocols. Furthermore, only upper extremity muscle strength was evaluated through handgrip tests, and no data on lower extremity muscle strength were reported. In addition, in some of the cohorts considered by Dodds and colleagues,^6^ the evaluation of muscle strength was not the main aim of the study.

According to the recommendations by EWGSOP2,^2^ our findings underline the importance of screening for sarcopenia starting from adult age, in order to implement adequate strategies aimed at preventing negative outcomes later in life.^30^, ^31^ The EWGSOP2 algorithm may be practically translated through the use of the charts generated by this study. Indeed, these curves may allow identifying individuals at risk for sarcopenia at young age. In this regard, it is worth noting that currently available cut‐offs for muscle strength are not applicable to young and adult persons and may therefore not be used for the early identification of people who might be at risk for sarcopenia. For instance, a handgrip strength value of 28 kg in a 35‐year‐old man would be considered ‘normal’ according to the cut‐offs suggested by EWGSOP2^2^ and the Foundation for National Institutes of Health sarcopenia project.^32^ However, such a handgrip strength value would fall at around the 5th percentile for age and gender based on Lookup 7+ data. According to the EWGSOP2 algorithm, handgrip strength and 5 × STS tests are equally valid for the initial assessment of sarcopenia.^2^ Hence, the choice of a specific test should be based on the availability of equipment and trained assessors as well as on test person's preferences and limitations.^2^, ^7^ In cases when both tests are applied and the performance in one of them is poor, the person should be classified as low‐performing and be offered further evaluations.

Albeit dealing with a highly relevant issue, our study presents some limitations that need to be discussed. First, normative values for muscle strength were obtained cross‐sectionally. Curves and centiles should therefore not be used to monitor an individual's trajectory of muscle strength over time. In addition, the possibility exists that findings might be influenced by differences in the birth cohort. The unconventional setting in which the study was conducted might have influenced the results of muscle strength testing. Indeed, although muscle strength was measured according to standard protocols, people who decided to participate in the study were involved—before being assessed—in a variety of activities, such as walking, carrying bags, and eating. Such activities could have influenced the assessment. Limitations also include the lack of information about specific medical conditions (e.g. osteoarthritis and other musculoskeletal and neurological disorders impacting muscle strength generation) and medications. However, it may reasonably be excluded that acute and/or severe illnesses were present at the time of evaluation. Indeed, candidate participants had to reach exhibition places and shopping centres, which might have selected a relatively healthy and functionally competent population. Although efforts were made to enrol a heterogeneous population, no detailed information was collected on the sociodemographic characteristics of participants. Hence, potential selection biases cannot be ruled out. It should however be considered that the unconventional setting in which the investigation was carried out required the assessment be completed within a reasonable amount of time. Finally, the study sample only included Caucasian persons; thus, our findings and charts may not be applicable to other ethnic groups.

Apart from these limitations, the Lookup 7+ project offered the unique opportunity to measure upper and lower extremity muscle strength in a large and relatively unselected population across a wide age spectrum. The normative curves and centile values generated by our study may greatly facilitate the practical appraisal of muscle strength and allow the timely identification of people with or at risk for sarcopenia.

### The lookup 7+ study group is composed as follows

*Steering Committee*: F. Landi and R. Bernabei.

*Coordination*: E. Marzetti, R. Calvani, and L. Mariotti.

*Writing Panel*: A. M. Martone, E. D'Angelo, E. Serafini, A. Picca, V. Palmieri, M. Tosato, P. Abete, A. Cherubini, F. Corica, and S. Salini.

*Field investigators*: M. Bianco, D. Biscotti, V. Brandi, M. Broccatelli, L. Catalano, F. Ciciarello, M. D'Elia, M. T. Fuga, E. Ortolani, G. Savera, and M. B. Zazzara.

*Participants*: A. Almohani, F. Benvenuto, M. Bianchi, G. Bramato, R. Capitani, C. Celesti, G. Colloca, F. Cuccaro, L. Damato, E. Di Murru, S. Fabrizi, D. Fusco, A. Galliani, V. Galluzzo, S. Gervasi, R. Quarta, G. Modica, R. Monti, S. Orvieto, C. Pais, C. Pantanelli, S. Rocchi, D. Ronconi, E. Rota, F. Sollazzo, and M. Tritto.

## Funding

Carni Sostenibili, Danone Italia, Errekappa, Ferrarini, Italfarmaco, Italia Longeva, IVSI, Laborest, Yakult, Marche Region, Nutricia, ProAction, Serenissima, and Novartis supported the Lookup 7+ project. The study was also partly supported by intramural research grants from the Università Cattolica del Sacro Cuore (D3.2 2013 and D3.2 2015) and by the non‐profit research foundation ‘Centro Studi Achille e Linda Lorenzon’.

## Author contributions

All of the authors participated in the conceiving, design, and writing of the manuscript. All authors read and approved the final manuscript.

## Conflict of interest

None to disclose.

## Supporting information


**Figure S1.** Reference percentiles of handgrip strength normalized by body mass index for men aged 18 to 80+ years. The 5^th^, 25^th^, 50^th^, 75^th^, and 95^th^ percentiles are shown in black, red, green, blue and purple, respectively.

**Figure S2.** Reference percentiles of handgrip strength normalized by body mass index for women aged 18 to 80+ years. The 5^th^, 25^th^, 50^th^, 75^th^, and 95^th^ percentiles are shown in black, red, green, blue and purple, respectively.

**Figure S3.** Reference percentiles of the 5‐repetition chair‐stand test normalized by body mass index for men aged 18 to 80+ years. The 5^th^, 25^th^, 50^th^, 75^th^, and 95^th^ percentiles are shown in black, red, green, blue and purple, respectively.

**Figure S4.** Reference percentiles of the 5‐repetition chair‐stand test normalized by body mass index for women aged 18 to 80+ years. The 5^th^, 25^th^, 50^th^, 75^th^, and 95^th^ percentiles are shown in black, red, green, blue and purple, respectively.

**Table S1.** Normative values for handgrip strength normalized by body mass index in men, stratified by age.

**Table S2.** Normative values for handgrip strength normalized by body mass index in women, stratified by age.

**Table S3.** Normative values for the 5‐repetition chair‐stand test normalized by body mass index in men, stratified by age.

**Table S4.** Normative values for the 5‐repetition chair‐stand test normalized by body mass index in women, stratified by age.

## References

[1] Anker SD , Morley JE , von Haehling S . Welcome to the ICD‐10 code for sarcopenia. J Cachexia Sarcopenia Muscle 2016;7:512–514. 27891296 10.1002/jcsm.12147 PMC5114626

[2] Cruz‐Jentoft AJ , Bahat G , Bauer J , Boirie Y , Bruyère O , Cederholm T , et al. Sarcopenia: revised European consensus on definition and diagnosis. Age Ageing 2019;48:16–31. 30312372 10.1093/ageing/afy169 PMC6322506

[3] Landi F , Calvani R , Tosato M , Martone AM , Fusco D , Sisto A , et al. Age‐related variations of muscle mass, strength, and physical performance in community‐dwellers: results from the Milan EXPO survey. J Am Med Dir Assoc 2017;18:88.e17–e24. 10.1016/j.jamda.2016.10.007 27914849

[4] Marzetti E , Hwang AC , Tosato M , Peng LN , Calvani R , Picca A , et al. Age‐related changes of skeletal muscle mass and strength among Italian and Taiwanese older people: results from the Milan EXPO 2015 survey and the I‐Lan Longitudinal Aging Study. Exp Gerontol 2018;102:76–80. 29246506 10.1016/j.exger.2017.12.008

[5] Sayer AA , Syddall H , Martin H , Patel H , Baylis D , Cooper C . The developmental origins of sarcopenia. J Nutr Health Aging 2008;12:427–432. 18615224 10.1007/BF02982703 PMC2652119

[6] Dodds RM , Syddall HE , Cooper R , Benzeval M , Deary IJ , Dennison EM , et al. Grip strength across the life course: normative data from twelve British studies. PLoS One 2014;9:e113637. 25474696 10.1371/journal.pone.0113637 PMC4256164

[7] Beaudart C , Rolland Y , Cruz‐Jentoft AJ , Bauer JM , Sieber C , Cooper C , et al. Assessment of muscle function and physical performance in daily clinical practice: a position paper endorsed by the European Society for Clinical and Economic Aspects of Osteoporosis, Osteoarthritis and Musculoskeletal Diseases (ESCEO). Calcif Tissue Int. 2019;105:1–14. 30972475 10.1007/s00223-019-00545-w

[8] Yeung SSY , Reijnierse EM , Trappenburg MC , Hogrel JY , McPhee JS , Piasecki M , et al. Handgrip strength cannot be assumed a proxy for overall muscle strength. J Am Med Dir Assoc 2018;19:703–709. 29935982 10.1016/j.jamda.2018.04.019

[9] Onder G , Penninx BW , Ferrucci L , Fried LP , Guralnik JM , Pahor M . Measures of physical performance and risk for progressive and catastrophic disability: results from the women's health and aging study. J Gerontol A Biol Sci Med Sci 2005;60:74–79. 15741286 10.1093/gerona/60.1.74

[10] Wang DXM , Yao J , Zirek Y , Reijnierse EM , Maier AB . Muscle mass, strength, and physical performance predicting activities of daily living: a meta‐analysis. J Cachexia Sarcopenia Muscle 2020;11:3–25. 31788969 10.1002/jcsm.12502 PMC7015244

[11] Landi F , Calvani R , Picca A , Tosato M , Martone AM , Ortolani E , et al. Cardiovascular health metrics, muscle mass and function among Italian community‐dwellers: the Lookup 7+ project. Eur J Public Health 2018;28:766–772. 29554257 10.1093/eurpub/cky034

[12] Vetrano DL , Martone AM , Mastropaolo S , Tosato M , Colloca G , Marzetti E , et al. Prevalence of the seven cardiovascular health metrics in a Mediterranean country: results from a cross‐sectional study. Eur J Public Health 2013;23:858–862. 24078648 10.1093/eurpub/ckt130

[13] von Elm E , Altman DG , Egger M , Pocock SJ , Gøtzsche PC , Vandenbroucke JP , et al. The Strengthening the Reporting of Observational Studies in Epidemiology (STROBE) statement: guidelines for reporting observational studies. Int J Surg 2014;12:1495–1499. 25046131 10.1016/j.ijsu.2014.07.013

[14] Marzetti E , Cesari M , Calvani R , Msihid J , Tosato M , Rodriguez‐Mañas L , et al. SPRINTT Consortium. The “Sarcopenia and Physical fRailty IN Older People: multi‐componenT Treatment Strategies” (SPRINTT) randomized controlled trial: case finding, screening and characteristics of eligible participants. Exp Gerontol 2018;113:48–57. 30261246 10.1016/j.exger.2018.09.017

[15] Bourgeois B , Fan B , Johannsen N , Gonzalez MC , Ng BK , Sommer MJ , et al. Improved strength prediction combining clinically available measures of skeletal muscle mass and quality. J Cachexia Sarcopenia Muscle 2019;10:84–94. 30371008 10.1002/jcsm.12353 PMC6438415

[16] Kuo Y‐L . The influence of chair seat height on the performance of community‐dwelling older adults' 30‐second chair stand test. Aging Clin Exp Res 2013;25:305–309. 23740582 10.1007/s40520-013-0041-x

[17] Landi F , Salini S , Zazzara MB , Martone AM , Fabrizi S , Bianchi M , et al. Relationship between pulmonary function and physical performance among community‐living people: results from Look‐up 7+ study. J Cachexia Sarcopenia Muscle 2020;11:38–45. 31800168 10.1002/jcsm.12485 PMC7015242

[18] Buckinx F , Landi F , Cesari M , Fielding RA , Visser M , Engelke K , et al. Pitfalls in the measurement of muscle mass: a need for a reference standard. J Cachexia Sarcopenia Muscle 2018;9:269–278. 29349935 10.1002/jcsm.12268 PMC5879987

[19] Cole TJ , Green PJ . Smoothing reference centile curves: the LMS method and penalized likelihood. Stat Med 1992;11:1305–1319. 1518992 10.1002/sim.4780111005

[20] Cole TJ , Bellizzi MC , Flegal KM , Dietz WH . Establishing a standard definition for child overweight and obesity worldwide: international survey. BMJ 2000;320:1240–1243. 10797032 10.1136/bmj.320.7244.1240 PMC27365

[21] Liu J , Yan Y , Xi B , Huang G , Mi J . Skeletal muscle reference for Chinese children and adolescents. J Cachexia Sarcopenia Muscle 2019;10:155–164. 30499245 10.1002/jcsm.12361 PMC6438334

[22] Landi F , Calvani R , Tosato M , Martone AM , Picca A , Ortolani E , et al. Animal‐derived protein consumption is associated with muscle mass and strength in community‐dwellers: results from the Milan EXPO survey. J Nutr Health Aging 2017;21:1050–1056.2908344710.1007/s12603-017-0974-4

[23] Landi F , Calvani R , Cesari M , Tosato M , Martone AM , Bernabei R , et al. Sarcopenia as the biological substrate of physical frailty. Clin Geriatr Med 2015;31:367–374. 26195096 10.1016/j.cger.2015.04.005

[24] Spruit MA , Sillen MJ , Groenen MT , Wouters EF , Franssen FM . New normative values for handgrip strength: results from the UK Biobank. J Am Med Dir Assoc. 2013;14:775.e5–775.e11. 10.1016/j.jamda.2013.06.013 23958225

[25] Leong DP , Teo KK , Rangarajan S , Kutty VR , Lanas F , Hui C , et al. Reference ranges of handgrip strength from 125,462 healthy adults in 21 countries: a prospective urban rural epidemiologic (PURE) study. J Cachexia Sarcopenia Muscle. 2016;7:535–546. 27104109 10.1002/jcsm.12112 PMC4833755

[26] Zengin A , Pye SR , Cook MJ , Adams JE , Rawer R , Wu FCW , et al. Associations of muscle force, power, cross‐sectional muscle area and bonegeometry in older UK men. J Cachexia Sarcopenia Muscle 2017;8:598–606. 28474432 10.1002/jcsm.12198 PMC5566651

[27] Molenaar HMT , Selles RW , Zuidam JM , Willemsen SP , Stam HJ . Growth diagrams for grip strength in children. Clin Orthop Relat Res 2010;468:217–223. 19459024 10.1007/s11999-009-0881-z PMC2795831

[28] Mathiowetz V , Wiemer DM , Federman SM . Grip and pinch strength: norms for 6‐ to 19‐year‐olds. Am J Occ Ther 1985;40:705–711. 10.5014/ajot.40.10.705 3777107

[29] De Smet L , Vercammen A . Grip strength in children. J Pedr Ortho B 2001;10:352–354. 11727383

[30] Martone AM , Bianchi L , Abete P , Bellelli G , Bo M , Cherubini A , et al. The incidence of sarcopenia among hospitalized older patients: results from the Glisten study. J Cachexia Sarcopenia Muscle 2017;8:907–914. 28913934 10.1002/jcsm.12224 PMC5700449

[31] Bauer J , Morley JE , Schols AMWJ , Ferrucci L , Cruz‐Jentoft AJ , Dent E , et al. Sarcopenia: a time for action. An SCWD position paper. J Cachexia Sarcopenia Muscle 2019;10:956–961. 31523937 10.1002/jcsm.12483 PMC6818450

[32] Studenski SA , Peters KW , Alley DE , Cawthon PM , McLean RR , Harris TB , et al. The FNIH sarcopenia project: rationale, study description, conference recommendations, and final estimates. J Gerontol A Biol Sci Med Sci 2014;69:547–558. 24737557 10.1093/gerona/glu010 PMC3991146

[33] von Haehling S , Morley JE , Coats AJS , Anker SD . Ethical guidelines for publishing in the Journal of Cachexia, Sarcopenia and Muscle: update 2019. J Cachexia Sarcopenia Muscle 2019;10:1143–1145. 31661195 10.1002/jcsm.12501 PMC6818444

