# Supplementary figures and images for: Normative values of muscle strength across ages in a ‘real world’ population: results from the longevity check‐up 7+ project
Source: J Cachexia Sarcopenia Muscle. 2020 Nov 4;11(6):1562–9. doi: 10.1002/jcsm.12610 (PMC7749608; doi:10.1002/jcsm.12610)

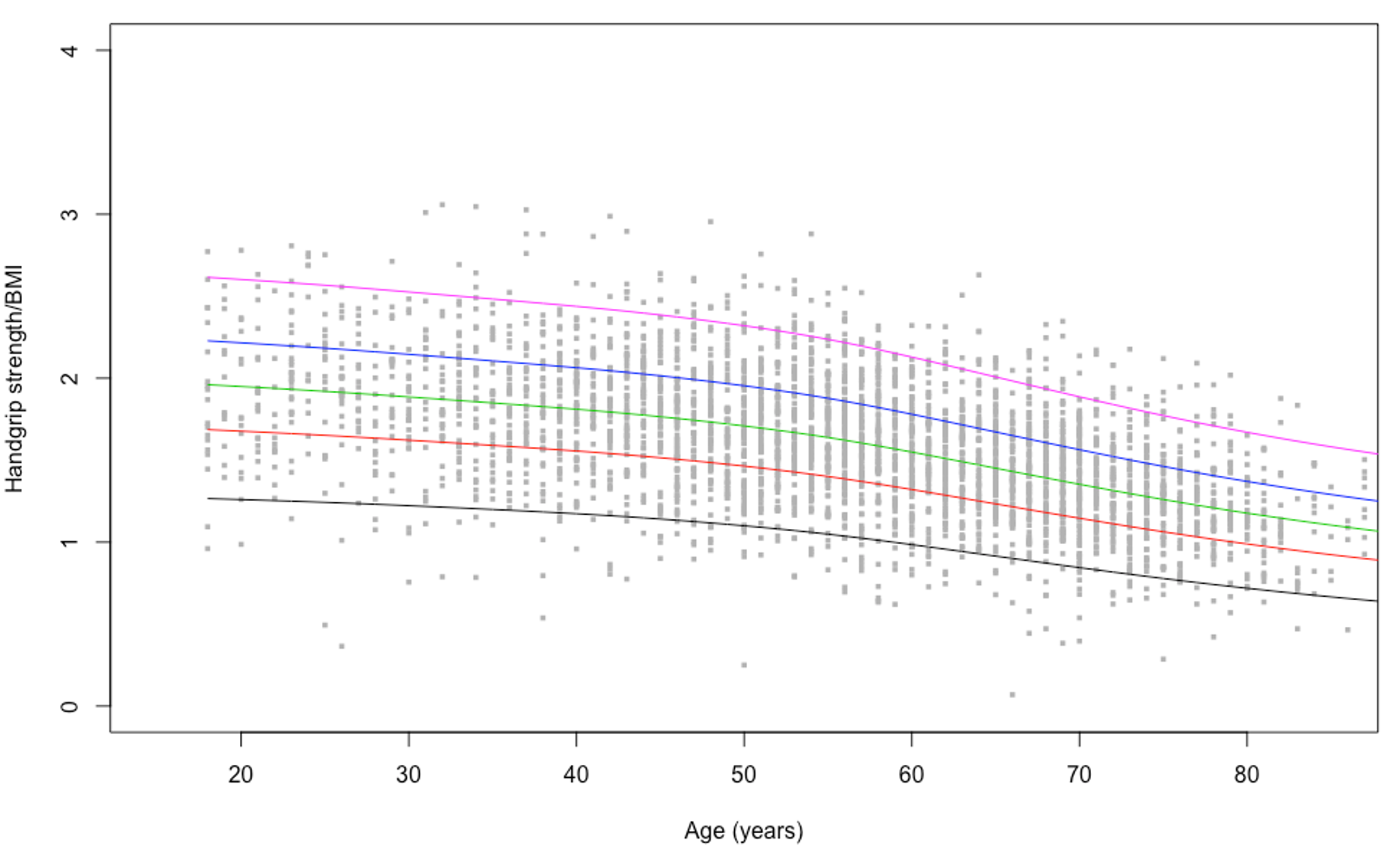

Supplement: Supplementary file 1 — Figure S1. Reference percentiles of handgrip strength normalized by body mass index for men aged 18 to 80+ years. The 5th, 25th, 50th, 75th, and 95th percentiles are shown in black, red, green, blue and purple, respectively. [file JCSM-11-1562-s001.tif]

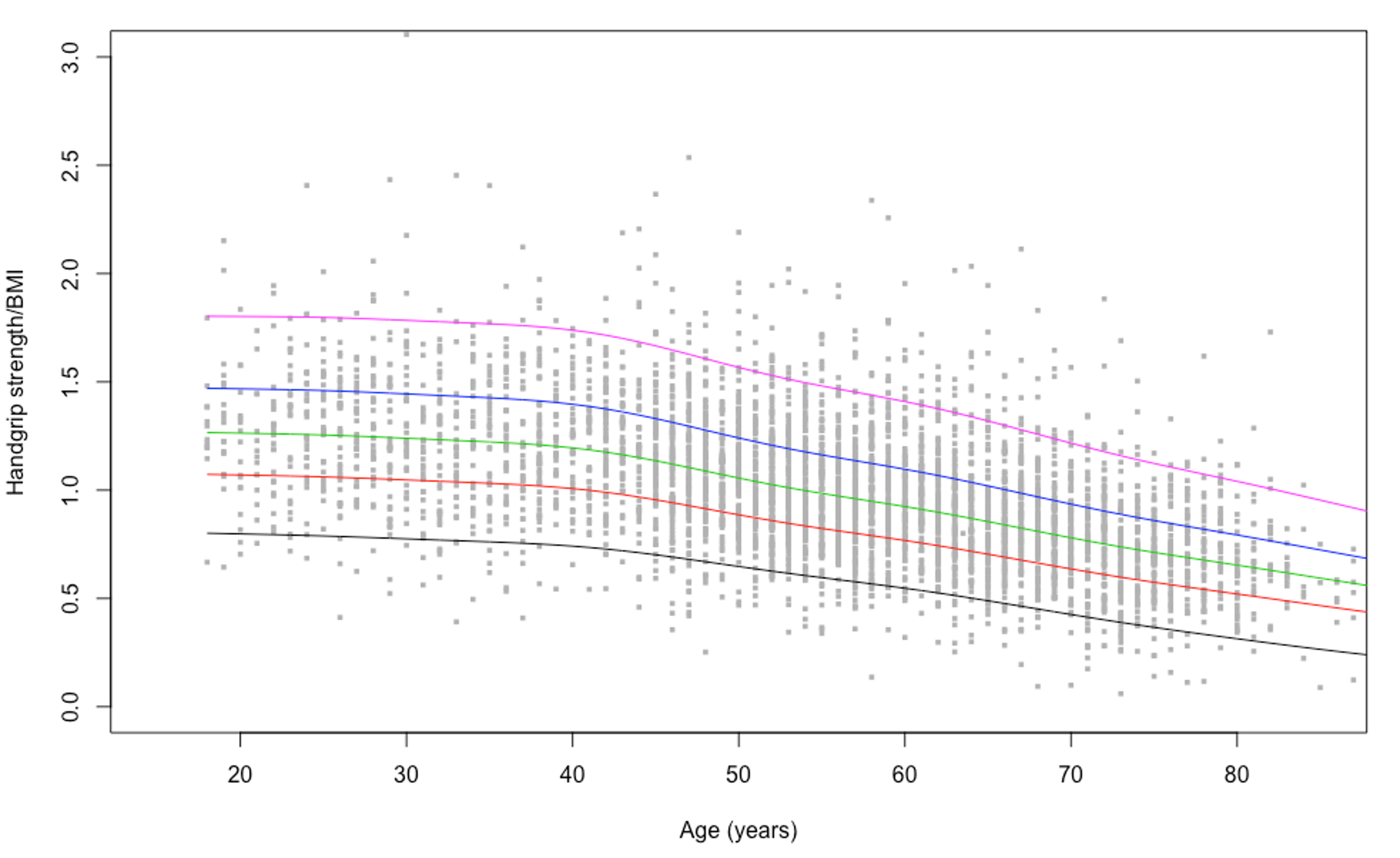

Supplement: Supplementary file 2 — Figure S2. Reference percentiles of handgrip strength normalized by body mass index for women aged 18 to 80+ years. The 5th, 25th, 50th, 75th, and 95th percentiles are shown in black, red, green, blue and purple, respectively. [file JCSM-11-1562-s002.tif]

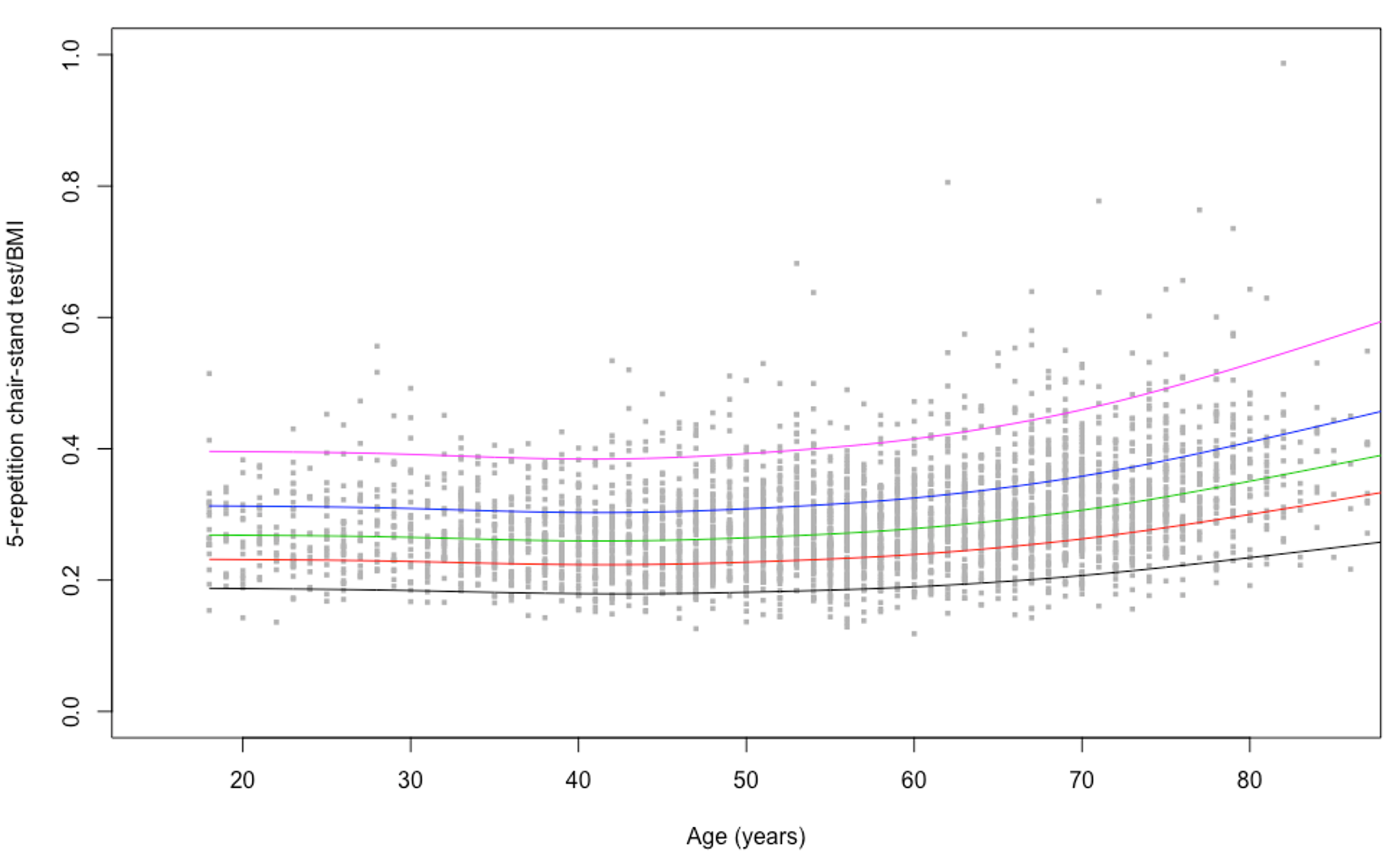

Supplement: Supplementary file 3 — Figure S3. Reference percentiles of the 5‐repetition chair‐stand test normalized by body mass index for men aged 18 to 80+ years. The 5th, 25th, 50th, 75th, and 95th percentiles are shown in black, red, green, blue and purple, respectively. [file JCSM-11-1562-s003.tif]

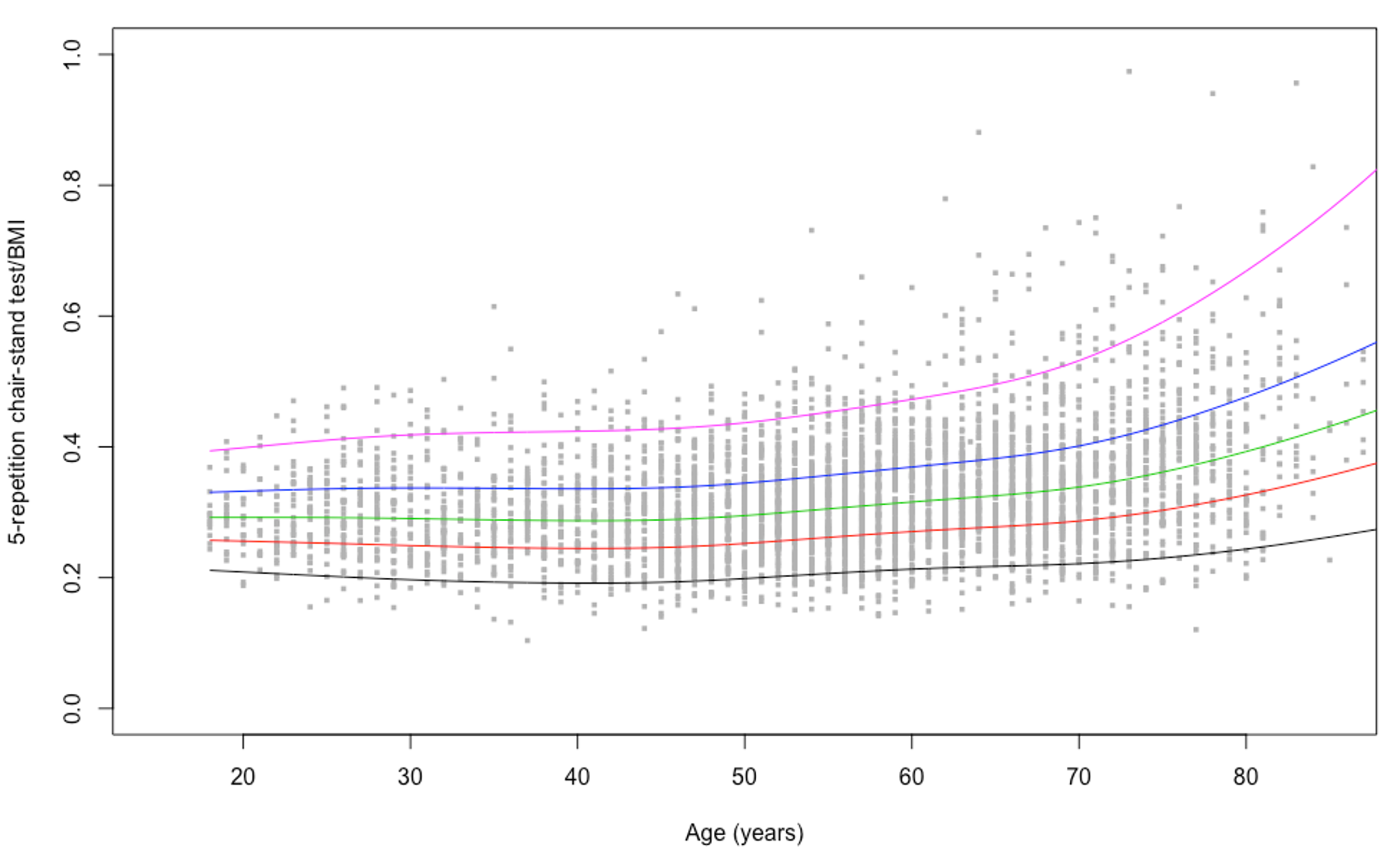

Supplement: Supplementary file 4 — Figure S4. Reference percentiles of the 5‐repetition chair‐stand test normalized by body mass index for women aged 18 to 80+ years. The 5th, 25th, 50th, 75th, and 95th percentiles are shown in black, red, green, blue and purple, respectively. [file JCSM-11-1562-s004.tif]
