# Supplementary material for: Normative values of muscle strength across ages in a ‘real world’ population: results from the longevity check‐up 7+ project
Source: J Cachexia Sarcopenia Muscle. 2020 Nov 4;11(6):1562–9. doi: 10.1002/jcsm.12610 (PMC7749608; doi:10.1002/jcsm.12610)
Supplement: Supplementary file 5 — Table S1. Normative values for handgrip strength normalized by body mass index in men, stratified by age. [file JCSM-11-1562-s005.docx]

**Table S1.** Normative values for handgrip strength normalized by body mass index in men, stratified by age.

| **Age groups (years)** | **Observations (n)** | **Centiles** | | | | | **Mean (standard deviation)** |
| --- | --- | --- | --- | --- | --- | --- | --- |
|  |  | **5^th^** | **25^th^** | **50^th^** | **75^th^** | **95^th^** |  |
| 18-24 | 146 | 1.340 | 1.634 | 1.921 | 2.226 | 2.688 | 1.940 (0.421) |
| 25-29 | 139 | 1.244 | 1.700 | 1.912 | 2.131 | 2.441 | 1.889 (0.412) |
| 30-34 | 186 | 1.172 | 1.616 | 1.865 | 2.131 | 2.481 | 1.869 (0.418) |
| 35-39 | 270 | 1.250 | 1.561 | 1.789 | 2.053 | 2.385 | 1.809 (0.379) |
| 40-44 | 369 | 1.236 | 1.583 | 1.832 | 2.062 | 2.356 | 1.819 (0.363) |
| 45-49 | 455 | 1.125 | 1.449 | 1.730 | 1.987 | 2.377 | 1.731 (0.377) |
| 50-54 | 598 | 1.092 | 1.419 | 1.669 | 1.938 | 2.304 | 1.685 (0.373) |
| 55-59 | 639 | 1.024 | 1.385 | 1.604 | 1.864 | 2.198 | 1.617 (0.361) |
| 60-64 | 547 | 0.972 | 1.286 | 1.497 | 1.730 | 2.090 | 1.514 (0.337) |
| 65-69 | 552 | 0.931 | 1.196 | 1.406 | 1.619 | 1.961 | 1.411 (0.325) |
| 70-74 | 503 | 0.829 | 1.104 | 1.302 | 1.544 | 1.807 | 1.322 (0.316) |
| 75-79 | 329 | 0.763 | 1.064 | 1.226 | 1.428 | 1.777 | 1.239 (0.306) |
| 80+ | 208 | 0.714 | 0.942 | 1.107 | 1.296 | 1.569 | 1.118 (0.283) |
| All | 4949 | 0.939 | 1.293 | 1.575 | 1.865 | 2.286 | 1.586 (0.416) |
