# Supplementary material for: Normative values of muscle strength across ages in a ‘real world’ population: results from the longevity check‐up 7+ project
Source: J Cachexia Sarcopenia Muscle. 2020 Nov 4;11(6):1562–9. doi: 10.1002/jcsm.12610 (PMC7749608; doi:10.1002/jcsm.12610)
Supplement: Supplementary file 6 — Table S2. Normative values for handgrip strength normalized by body mass index in women, stratified by age. [file JCSM-11-1562-s006.docx]

**Table S2.** Normative values for handgrip strength normalized by body mass index in women, stratified by age.

| **Age groups (years)** | **Observations (n)** | **Centiles** | | | | | **Mean (standard deviation)** |
| --- | --- | --- | --- | --- | --- | --- | --- |
|  |  | **5^th^** | **25^th^** | **50^th^** | **75^th^** | **95^th^** |  |
| 18-24 | 181 | 0.784 | 1.067 | 1.260 | 1.446 | 1.807 | 1.273 (0.313) |
| 25-29 | 248 | 0.827 | 1.060 | 1.262 | 1.481 | 1.731 | 1.269 (0.303) |
| 30-34 | 234 | 0.748 | 0.990 | 1.204 | 1.413 | 1.706 | 1.227 (0.351) |
| 35-39 | 280 | 0.732 | 1.022 | 1.205 | 1.436 | 1.711 | 1.226 (0.313) |
| 40-44 | 345 | 0.800 | 1.021 | 1.220 | 1.383 | 1.661 | 1.217 (0.282) |
| 45-49 | 633 | 0.641 | 0.927 | 1.099 | 1.278 | 1.578 | 1.110 (0.298) |
| 50-54 | 919 | 0.644 | 0.853 | 1.011 | 1.204 | 1.514 | 1.043 (0.271) |
| 55-59 | 839 | 0.567 | 0.784 | 0.949 | 1.141 | 1.460 | 0.980 (0.284) |
| 60-64 | 786 | 0.539 | 0.746 | 0.917 | 1.066 | 1.403 | 0.927 (0.264) |
| 65-69 | 758 | 0.497 | 0.687 | 0.828 | 0.984 | 1.285 | 0.850 (0.255) |
| 70-74 | 563 | 0.367 | 0.597 | 0.744 | 0.901 | 1.169 | 0.754 (0.249) |
| 75-79 | 395 | 0.357 | 0.553 | 0.697 | 0.853 | 1.054 | 0.707 (0.223) |
| 80+ | 192 | 0.285 | 0.500 | 0.615 | 0.753 | 1.016 | 0.630 (0.239) |
| All | 6382 | 0.504 | 0.763 | 0.958 | 1.191 | 1.562 | 0.991 (0.328) |
