# Supplementary material for: Normative values of muscle strength across ages in a ‘real world’ population: results from the longevity check‐up 7+ project
Source: J Cachexia Sarcopenia Muscle. 2020 Nov 4;11(6):1562–9. doi: 10.1002/jcsm.12610 (PMC7749608; doi:10.1002/jcsm.12610)
Supplement: Supplementary file 7 — Table S3. Normative values for the 5‐repetition chair‐stand test normalized by body mass index in men, stratified by age. [file JCSM-11-1562-s007.docx]

**Table S3.** Normative values for the 5-repetition chair-stand test normalized by body mass index in men, stratified by age.

| **Age groups (years)** | **Observations (n)** | **Centiles** | | | | | **Mean (standard deviation)** |
| --- | --- | --- | --- | --- | --- | --- | --- |
|  |  | **5^th^** | **25^th^** | **50^th^** | **75^th^** | **95^th^** |  |
| 18-24 | 146 | 0.188 | 0.229 | 0.263 | 0.314 | 0.372 | 0.273 (0.062) |
| 25-29 | 136 | 0.195 | 0.234 | 0.273 | 0.324 | 0.429 | 0.287 (0.074) |
| 30-34 | 186 | 0.193 | 0.229 | 0.261 | 0.315 | 0.388 | 0.274 (0.064) |
| 35-39 | 268 | 0.185 | 0.227 | 0.257 | 0.296 | 0.375 | 0.264 (0.056) |
| 40-44 | 367 | 0.172 | 0.220 | 0.264 | 0.300 | 0.397 | 0.267 (0.066) |
| 45-49 | 625 | 0.185 | 0.222 | 0.259 | 0.307 | 0.402 | 0.270 (0.066) |
| 50-54 | 910 | 0.184 | 0.235 | 0.273 | 0.311 | 0.403 | 0.283 (0.099) |
| 55-59 | 830 | 0.183 | 0.230 | 0.270 | 0.313 | 0.376 | 0.274 (0.061) |
| 60-64 | 772 | 0.190 | 0.247 | 0.286 | 0.336 | 0.428 | 0.295 (0.076) |
| 65-69 | 747 | 0.199 | 0.254 | 0.297 | 0.351 | 0.455 | 0.308 (0.079) |
| 70-74 | 547 | 0.207 | 0.264 | 0.310 | 0.361 | 0.454 | 0.319 (0.080) |
| 75-79 | 377 | 0.235 | 0.293 | 0.337 | 0.398 | 0.506 | 0.353 (0.091) |
| 80+ | 178 | 0.231 | 0.307 | 0.376 | 0.422 | 0.554 | 0.377 (0.110) |
| All | 4893 | 0.189 | 0.239 | 0.282 | 0.333 | 0.433 | 0.293 (0.082) |
