# Supplementary material for: Normative values of muscle strength across ages in a ‘real world’ population: results from the longevity check‐up 7+ project
Source: J Cachexia Sarcopenia Muscle. 2020 Nov 4;11(6):1562–9. doi: 10.1002/jcsm.12610 (PMC7749608; doi:10.1002/jcsm.12610)
Supplement: Supplementary file 8 — Table S4. Normative values for the 5‐repetition chair‐stand test normalized by body mass index in women, stratified by age. [file JCSM-11-1562-s008.docx]

**Table S4.** Normative values for the 5-repetition chair-stand test normalized by body mass index in women, stratified by age.

| **Age groups (years)** | **Observations (n)** | **Centiles** | | | | | **Mean (standard deviation)** |
| --- | --- | --- | --- | --- | --- | --- | --- |
|  |  | **5^th^** | **25^th^** | **50^th^** | **75^th^** | **95^th^** |  |
| 18-24 | 181 | 0.222 | 0.253 | 0.290 | 0.329 | 0.391 | 0.295 (0.056) |
| 25-29 | 244 | 0.194 | 0.243 | 0.293 | 0.341 | 0.424 | 0.299 (0.071) |
| 30-34 | 233 | 0.202 | 0.246 | 0.286 | 0.335 | 0.424 | 0.296 (0.069) |
| 35-39 | 277 | 0.196 | 0.253 | 0.288 | 0.331 | 0.437 | 0.296 (0.072) |
| 40-44 | 367 | 0.194 | 0.246 | 0.292 | 0.341 | 0.435 | 0.298 (0.073) |
| 45-49 | 453 | 0.195 | 0.240 | 0.283 | 0.342 | 0.415 | 0.294 (0.074) |
| 50-54 | 594 | 0.203 | 0.262 | 0.304 | 0.348 | 0.443 | 0.310 (0.079) |
| 55-59 | 637 | 0.202 | 0.263 | 0.310 | 0.366 | 0.455 | 0.317 (0.078) |
| 60-64 | 543 | 0.214 | 0.277 | 0.323 | 0.378 | 0.461 | 0.333 (0.083) |
| 65-69 | 547 | 0.216 | 0.279 | 0.327 | 0.387 | 0.493 | 0.342 (0.109) |
| 70-74 | 498 | 0.225 | 0.289 | 0.342 | 0.408 | 0.558 | 0.359 (0.107) |
| 75-79 | 322 | 0.237 | 0.316 | 0.387 | 0.444 | 0.598 | 0.403 (0.149) |
| 80+ | 189 | 0.242 | 0.329 | 0.424 | 0.504 | 0.733 | 0.436 (0.147) |
| All | 6271 | 0.205 | 0.265 | 0.313 | 0.371 | 0.484 | 0.236 (0.097) |
